# Supplementary material for: Demystifying Case Management in Aotearoa New Zealand: A Scoping and Mapping Review
Source: Int J Environ Res Public Health. 2022 Dec 31;20(1):784. doi: 10.3390/ijerph20010784 (PMC9819615; doi:10.3390/ijerph20010784)
Supplement: Supplementary file 1 [file ijerph-20-00784-s001.zip › ijerph-1986869-supplementary.pdf]

Table S1. Summary of data sources for each role

No.	Role	Health databases			Grey literature				Interviews	
		Article	Chapter	Theses	Reports Evaluations	Job Advert	Māori and Pacific websites	Other: Videos, presentation magazines	Interview	Ecomap
**1**	Whānau Ora Navigator	√√√√√	√	√	√√√√√√√√√√√	√	√√√√√√√√√√√√√√√√√√√√	√		
**2**	Kai Manaaki (KM)	√√√√			√√			√√√		
**3**	Māori Cancer Coordinator	√		√√			√√			
**4**	Pacific Navigator				√				√	√
**5**	Partnership Community Worker (PCW)	√		√				√√		
**6**	Case Manager (Private Insurance)				√	√√√√			√	√
**7**	ACC Recovery Coordinator					√	√√		√	√
**8**	ACC Recovery Partner	√√							√	√
**9**	Practice Nurse Case Manager	√√√√√√√√√<br>√			√√		√√		√	√
**10,11**	Behavioural Support Practitioner (Health Coach or Health Improvement Practiioner)				√	√		√√	√	√√
**12**	Needs Assessment Service Coordinator (NASC)				√	√√	√√√√√	√	√√	√√
**13**	Local Area Coordinator (LAC)				√√			√		
**14**	Kaitūhuno (Connector)				√			√√		
**15**	Care Manager Older Adults	√√√√√				√√√			√	√
**16**	Cancer Nurse Coordinator	√√			√					
**17**	Case Loading Midwives	√		√					√√	
**18**	Key Worker: Mental Health	√				√√√√√			√√	
